# The technological influence on health professionals' care: translation and adaptation of scales^1^

**DOI:** 10.1590/1518-8345.0990.2681

**Published:** 2016-05-03

**Authors:** Carlos Manuel Torres Almeida, Filipe Nuno Alves dos Santos Almeida, Joaquim José Jacinto Escola, Vitor Manuel Costa Pereira Rodrigues

**Affiliations:** 2 PhD, Adjunct Professor, Centro de Investigação em Desporto, Saúde e Desenvolvimento Humano, Escola Superior de Enfermagem de Vila Real, Universidade de Trás-os-Montes e Alto Douro, Vila Real, Portugal; 3 PhD, Assistant Professor, Departamento de Educação e Simulação Médica, Faculdade de Medicina, Universidade do Porto, Porto, Portugal; 4 PhD, Assistant Professor, Escola das Ciências Humanas e Socias, Universidade de Trás-os-Montes e Alto Douro, Vila Real, Portugal; 5 PhD, Coordinator Professor, Centro de Investigação em Desporto, Saúde e Desenvolvimento Humano, Escola Superior de Enfermagem de Vila Real, Universidade de Trás-os-Montes e Alto Douro, Vila Real, Portugal

**Keywords:** Technological Development, Delivery of Health Care, Scales

## Abstract

**Objectives:**

in this study, two research tools were validated to study the impact of technological influence on health professionals' care practice.

**Methods:**

the following methodological steps were taken: bibliographic review, selection of the scales, translation and cultural adaptation and analysis of psychometric properties.

**Results:**

the psychometric properties of the scale were assessed based on its application to a sample of 341 individuals (nurses, physicians, final-year nursing and medical students). The validity, reliability and internal consistency were tested. Two scales were found: Caring Attributes Questionnaire (adapted) with a Cronbach's Alpha coefficient of 0.647 and the Technological Influence Questionnaire (adapted) with an Alpha coefficient of 0.777.

**Conclusions:**

the scales are easy to apply and reveal reliable psychometric properties, an additional quality as they permit generalized studies on a theme as important as the impact of technological influence in health care.

## Introduction

The contemporary world is clearly technological, entailing advances in many of the areas that affect human life. In that sense, health care may represent the area where these advances are more visible and provoke greater expectations. Nevertheless, this technological progress "has aroused different concerns and questions on the benefits, risks and the relations constructed among workers, patients and technology use"^(^ 1 ^)^ **_._** Some even appoint that, together with this scientific progress, the dissatisfaction with health professionals seems to increase. At the same time as the scientific knowledge and new treatment and diagnostic techniques evolve, the patients' dissatisfaction with health care grows, which seems to point towards difficulties to achieve a harmonious relation between scientific progress and the prioritization of what is human in health care.

In several countries, today, professional organizations elaborate large-scale opinions and studies to defend the users and the quality of health care, with outcomes that are highly critical of health professionals and seem to want to alert to the fact that these workers have lost their ability to "take care"^(^ 2 ^-^ 5 ^)^.

One of the most appointed aspects in the bibliography is the excessive technicality of man's action in the 21^st^ century, or the extreme rationalization of the contemporary technical civilization which, according to Silva and Ferreira^(^ 6 ^)^, exerts cultural and social control on human beings, sometimes leading to the rational automatism that replaces individual and group decision making. This attitude, associated with the strong influence of the so-called "biomedical model", strongly in vogue in the health care of the past centuries, may have lead to a "mix-up" of values which, according to the authors, managed to deviate the health practices from their core objective, which is the human being. When centering almost exclusively on the diagnosis, disease and treatment forms, the health professionals often leave ill people in the hands of a depersonalizing solitude, a fact that irreparably impairs the quality of health care, affecting it exactly in one of its paramount characteristics, which is the therapeutic relationship or the relationship patient/health professional.

The patients' dissatisfaction with health care or with the health professionals' attitudes can make access to care more difficult, as fear and dislike can lead to distancing between patient and health institutions.

Thus, some questions emerge: is technological development an obstacle for the implementation of high-quality and patient-centered health care? What is the effect of the increased technological influence on the health professionals' performance? Can technology make care more efficient, thus facilitating the patients' access to health care?

In 1997, in one study^(^ 7 ^)^, the participants (14 nurses) displayed a positive view on the benefits of technology and their trust in the potential the "machine" offered. In addition, in the study Describing the Influence of Technologies on Registered Nurses' Work^(^ 8 ^)^, this professional group's valuation of technology was revealed. In their perspective, technology encourages more efficient practice and helps to save time, thus eliminating wasted efforts, improving service and enhancing the patients' safety. At bottom, in that study, the following are appointed as aspects that need to be valued in the technology: the fact that technology improves care delivery, improves the outcomes for the patient, improves practice and improves the care environment.

In that sense, the study The effect of technology on the caring attributes of an international samples of nurses^(^ 9 ^)^ is particularly noteworthy, as it raises the possibility of a new approach to this problem, which had thus far only been discussed based on phenomenological or qualitative methods.

In view of these findings, the translation, adaptation and validation of two data collection tools - TIQ (Technological Influence Questionnaire) and CAQ (Caring Attributes Questionnaire), seems to be very useful to start a study on this theme. Although these tools have only been applied to Nursing professionals, according to the authors, as the technological influence and its possible effects on care will certainly affect all health care providers, it would be interesting to apply those tools to various professional groups in that are. Nevertheless, due to difficulties to get access to some professional groups for sampling purposes, according to established tool validation rules, in this study, the researchers chose to apply the tools to nursing professionals, medical professionals, nursing students and medical students only was chosen. Therefore, some items in the scales were slightly adapted.

## Method

### Caring Attributes Questionnaire (CAQ)

The introduction of scales that permit the quantitative assessment of caring attributes is very recent. Great efforts in that sense only started to be made as from the 1980's. The Caring Attributes Questionnaire (CAQ)^(^ 9 ^)^ has been used in different countries on distinct continents, with very solid psychometric characteristics, easy comprehension and completion. The tool uses an agreement scale and consists of 31 items, grouped in four subscales: care communication; caring as advocacy; engagement in care and learning to care. 

### Technological Influence Questionnaire (TIQ)

The Technological Influence Questionnaire^(^ 9 ^)^ is a single-factor tool that consists of 14 items, using an agreement scale.

The CAQ and TIQ are applied individually and, although originally directed at Nursing professionals, adaptations were made in this study to be applicable not only to nursing professionals, but also to medical professionals and nursing students and medical students.

### Scale translation and validation procedures

The translation and adaptation of scales require strict procedures that go far beyond simple translation. The cultural contexts need to be heeded, whether of the original culture or the target culture of the test, thus implying not only the translation, but a global adaptation to the new situation. Thus, the goal is for the test to similarly measure the original construct, even if that demands adjustments to the particularities of the study population^(^ 10 ^-^ 11 ^)^ *_._*

In line with research experts' orientations, the CAQ and TIQ were translated to Portuguese in five steps. In the first, two bilingual professionals translated the scale from the original language, in this case English, to Portuguese. These experts were asked to use simple language but to, beyond a literal translation, attempt to capture the meaning of the different items. After the individual translations, they were asked to analyze both translations and solve the discrepancies found in order to elaborate a single document.

After this phase, two other bilingual experts elaborated the back translation^(^ 11 ^)^ **_,_** then comparing the results. Next, two experts fluent in English developed an independent review. They were knowledgeable on the study objectives and the target population and were asked to compare the back-translated version (in English) with the original scale.

To solve possible difficulties in the understanding of some of the items, a pretest was applied to 12 individuals from the health area (nursing and medical professionals and students).

### Validation procedures and criteria of the CAQ and TIQ scales

To assess the psychometric properties of the scales and analyze the results of their application, maintaining the author's method was considered to be most correct to make it easier to compare the results. Hence, after inverting the scores of items that were formulated in the opposite sense, validity and reliability tests were applied, based on a set of criteria that follow the best practices. Thus, the data resulting from exploratory factor analysis were crossed with Cronbach's alpha and item-item and item-total correlation coefficients. In that sense, the following criteria were set^(^ 12 ^)^.

- For the factor analysis, the principal component extraction method was used, adopting four main components to respect the structural organization of the original scale, followed by the rotation of the factors to obtain a clearer and more objective factor solution, thus maximizing the factor loadings of the items^(^ 12 ^)^. Like the scale author, the researchers chose the Varimax rotation method.

To determine what factors and items to retain, different authors' recommendations were followed^(^ 12 ^-^ 15 ^)^: a) Kaiser criterion - factors with eigenvalue of 1 or higher (EV≥1); b) factor loadings of items equal to or higher than 0.3 (FL≥0,30); although several authors suggest higher loadings of 0.5, the researchers again considered that the original scale author's criteria should be maintained; c) inexistence of items with relevant factor ladings (superior to 0.30) in more than one factor. If that happens and if the difference between them is not equal to or higher than 0.15, the elimination of the item should be considered; d) the percentage of the variance the retained factors explain should be at least 40% and e) each factor can contain no less than three items.

To complement the reliability analysis, 0.60 was set as the minimal internal consistency ratio (Cronbach's alpha), the item/item-total correlation should not be lower than 0.3 and the internal consistency of the factor should not increase if the item were eliminated.

## Results

Considering the preset inclusion criteria and to reduce the universe of care providers to be included in the target population, stratified sampling was chosen to obtain a representative sample, according to some pre-identified variables of the study population and non-probabilistic convenience - snowball - sampling. Hence, the data were collected online. The data collection tool was available between June and December 2012, and the participants received an e-mail with the website to complete the tools.

As regards the ethical procedures, participation in the study was voluntary. The confidentiality of the results and the respondents' anonymity were guaranteed. The Ethics Committee from a Hospital Center in Northern Portugal approved the study on 06/21/2012.

The sample consisted of 342 individuals, distributed as follows: 40.4% of the sample were Nursing professionals (138), 31.3% (107) are final-year students from the graduation course in Nursing, 15.8% (54) are physicians and 12.6% (43) are final-years students from the Medicine program.

### Caring Attributes Questionnaire (CAQ)

To serve as a suitability criterion for the factor analysis, Kaiser-Meyer-Olkin (KMO) and Bartlett's sphericity tests were applied. The KMO coefficient of 0.882 suggests, according to the literature, that the application of FA is clearly recommendable (coefficients superior to 0.60 indicate that the analysis is fit).

On the other hand, even if not that reliable, the significance of Bartlett's sphericity test (coefficients associated with p<0.05) shows that the variables can be correlated^(^ 13 ^)^.

Table 1 shows the results of an exploratory analysis using the principal component extraction method, revealing four principal components, aiming to respect the structural organization of the original scale, followed by the Varimax rotation method. As a whole, the intended four-factor organization justifies 41.685% of the total variance (factor 1-13.68; factor 2-11.121; factor 3-8.683 and factor 4-8.198).

**Table 1:** - Component matrix. Northern Portugal, Portugal, 2012

**Factor 1**	**Factor 2**	**Factor 3**	**Factor 4**				
**Item**	**Loading**	**Item**	**Loading**	**Item**	**Loading**	**Item**	**Loading**
P14	.596	P2	.340	P1	.373	P5	.521
P15	.633	P3	.316	P27	-.465	P10	.513
P17	.635	P4	.488	P28	.613	P13	.597
P18	.600	P6	.677	P29	.759	P16	.484
P19	.659	P7	.746	P30	.637	P22	-.484
P20	.605	P8	.673	P31	.753	P25	.542
P21	.477	P9	.347			P26	.659
P23	.662	P11	.656				
P24	.642	P12	.611				

The distribution of the items among the four factors obtained through the principal component analysis differs from the distribution found in the international sample and, therefore, differs from the author's proposed distribution. Nevertheless, the items belonging to each of the initial scales are clearly predominant. In addition, when applying the established criteria, some items should be eliminated.

Thus, factor 1 is composed of nine items: five items belonging to the original advocacy scale (19, 20, 21, 23 and 24), besides items 14, 15, 17 and 18, which theoretically belonged to the communication scale. Based on a semantic analysis of the content of these items, this divergence can be easily justified by cultural differences, since the professionals consider actions like respect for confidentiality or management of the information for family members more as a form of patient advocacy than as actual communication criteria. The saturation levels range from 0.477 for item 21 ("caring means talking on the patient's behalf ...") to 0.659 for item 19 ("caring means preventing possible complications").

In this study, factor 2 consists of nine items, seven of which belong to the communication scale (items 3, 4, 6, 7, 8, 11 and 12), item 2 - "caring is not significant for the patient's health condition", which belongs to the caring engagement scale, and item 9 -"caring means demonstrating professional competences", which originally belongs to the advocacy scale. The communication items, except for item 3 with a saturation level of 0.316, present high saturation levels (>0.600), the highest of which (0.746) is item 7 "I am caring when I talk to the patient".

Factor 3 contains six items, five of which belong to the care learning scale, almost all with high saturation levels (0.613; 0.759; 0.637 and 0.753), and item 27, which originally belongs to the care engagement scale. Finally, factor 4 contains six out of eight items that make up the original engagement scale (5, 10, 13, 16, 25 and 26), all with saturation levels ranging between 0.484 and 0.659, besides item 22 ("I am caring when I talk on the patient's behalf about his care"), which supposedly belongs to the advocacy scale.

### Reliability and internal consistency analysis

As the author of the original scale describes, Pearson's correlation coefficient is determined between each item and the scale it belongs to, knowing that, in the original study, items with r>0.3 were used as the inclusion criterion and Cronbach's alpha coefficient was determined.

For factor 1, for which the designation advocacy scale will be maintained and which consists of nine items, the alpha coefficient is -.834, higher than in the original study (0.78). The item/total correlation coefficients range between 0.352, corresponding to the question "caring means talking on the patient's behalf when the health professional perceives that something harmful (for the patient) might be done" and 0.640 for the question "caring means preventing possible complications".

For factor 2, for which the scale designation "care communication" was maintained, in the factor analysis, a nine-item structure was obtained. After the analysis revealed that items 2 and 9 were semantically inappropriate to the rest of the factor and that the communality values were low, together with an item/item-total correlation coefficient inferior to 0.3, against the author's own criteria, the decision was made to eliminate these items. Hence, for the scale that now consists of seven items, an alpha coefficient of 0.777 was obtained, lower than what the author obtained (0.89), but easily justifiable by the reduction in the number of items when compared to the original scale (from 11 to 7). Table 2 reveals that the item/total correlations range between 0.334 and 0.538, thus respecting the established criteria.

**Table 2:** - Item/total correlation and Cronbach's alpha reliability of four factors. Northern Portugal, Portugal, 2012

**Factor 1**	**Factor 2**	**Factor 3**	**Factor 4**				
**Item**	**r**	**Item**	**R**	**Item**	**r**	**Item**	**r**
P14	.488	P3	.327	P28	.406	P5	.404
P15	.556	P4	.448	P29	.569	P10	.318
P17	.529	P6	.495	P30	.433	P25	.450
P18	.507	P7	.561	P31	.569	P26	.541
P19	.640	P8	.505				
P20	.577	P11	.566				
P21	.374	P12	.482				
P23	.565						
P24	.553						
Cronbach's Alpha	Cronbach's Alpha	Cronbach's Alpha	Cronbach's Alpha				
0.834	0.777	0.709	0.647				

Concerning factor 3, the scale designation "care learning" was maintained. After careful analysis, item 27 was eliminated due to semantic inappropriateness, and item 1 because of r<0.3. Hence, for this scale, which now consists of four items, the alpha coefficient (0.709) is superior to what the author obtained in the international sample. As for the correlation coefficient, all other items presented r coefficients superior to 0.3.

Factor 4, which represents the "care engagement" scale, demonstrated results most distant from the author's, deserving some reflection. The analysis of the seven items obtained through the factor analysis revealed that item 22, because of its semantic content, is not fit for this dimension, and was therefore eliminated, while items 13 and 16 presented r coefficients <0.3, against the established criteria, and were therefore also eliminated. Hence, this dimension is now constituted of four items only, above the minimum set (three items), but substantially different from the original scale (eight items). This composition obtained an alpha coefficient of 0.647, significantly lower than the original (0.79), but once again justifiable by the reduction in the number of items.

Concerning the item/total correlation coefficients obtained, all coefficients comply with the preset criteria.

### Technological Influence Questionnaire (TIQ)

To serve as a suitability criterion for the factor analysis, Kaiser-Meyer-Olkin (KMO) and Bartlett's sphericity tests were applied. The KMO coefficient of 0.813 suggests, according to the literature, that the application of FA is clearly recommendable (coefficients superior to 0.60 indicate that the analysis is fit).

On the other hand, even if not that reliable, the significance of Bartlett's sphericity test (values associated with p<0.05) shows that the variables can be correlated^(^ 13 ^)^.

Although the scale author uses a one-factor structure in the different studies, the scale was submitted to exploratory factor analysis through the principal component extraction method, using the Kaiser criterion - factors with eigenvalue equal or superior to 1 (EV≥1), in order to verify whether this organization was confirmed. This factor analysis revealed that the items were distributed among three factors that explain 49.712% of the total variance (factor 1-22.250; factor 2-16.547 and factor 3-10.914).

Thus, the distribution of the items among the three factors obtained through the principal component analysis (Table 3) differs from the author's original idea but deserves more careful analysis. Hence, factor 1 emerges with six items (4, 6, 7, 8, 9 and 11), all of which are formulated in the negative form and require inverted scores. The saturation levels range between 0.390 for item 6 ("due to using more technology, the health professionals feel frustrated when a patient dies") and 0.760 for item 9 ("I am in doubt about the benefits of the technology for my (future) profession").

In this study, factor 2 emerges with four items (10, 12, 13 and 14), formulated in the positive sense to value the health technology. These items display high saturation levels, superior to 0.60.

In factor 3, four items are evidenced (1, 2, 3 and 5).

**Table 3:** - Component matrix. Northern Portugal, Portugal, 2012

**Factor 1**	**Factor 2**	**Factor 3**			
**Item**	**Loading**	**Item**	**Loading**	**Item**	**Loading**
P4	.684	P10	.629	P1	.702
P6	.390	P12	.706	P2	.424
P7	.679	P13	.655	P3	-.573
P8	.757	P14	.795	P5	.570
P9	.760				
P11	.726				

### Reliability and internal consistency analysis

As mentioned in the validity criteria for the reliability and internal consistency analyses, in this study, Cronbach's alpha coefficients were analyzed and Pearson's correlation coefficients between each item and the scale it belongs to were determined. Only item/total correlations with r>0.3 were considered.

From a careful analysis of the items, it was verified that item 6 not only presents a low communality value (0.220), but that the r-coefficient is inferior to the intended 0.3. Therefore, the item was excluded, which even increases the alpha coefficient.

For factor 1, for which the scale designation "negative influence" will be used, which now consists of five items, the alpha coefficient was 0.80, which is considered good. As for the item/total correlation, all coefficients are superior to 0.5.

For factor 2, for which the scale designation "positive influence" was maintained, in the factor analysis, a four-item structure was obtained. The detailed analysis of each item's behavior showed that all items comply with the preset criteria (Table 4). Hence, for this dimension, an alpha coefficient of 0.709 was obtained, which is low but which, according to several authors, is considered acceptable. In addition, dimensions with few items frequently obtain lower alpha coefficients. The table reveals that all item/total correlations range between 0.438 and 0.527.

Concerning factor 3, this association seemed difficult from the start. Thus, after a more careful analysis, it was verified that all items presented r-coefficients far below the established values. Therefore, it was decided to exclude these items.

**Table 4:** - Two-factor item/total correlation and Cronbach's alpha reliability. Northern Portugal, Portugal, 2012

**Factor 1**	**Factor 2**		
**Item**	**r**	**Item**	**r**
P4	.569	P10	.481
P7	.537	P12	.516
P8	.558	P13	.438
P9	.631	P14	.527
P11	.604		
Cronbach's Alpha	Cronbach's Alpha		
0.800	0.709		

## Discussion

**Figure 1:**
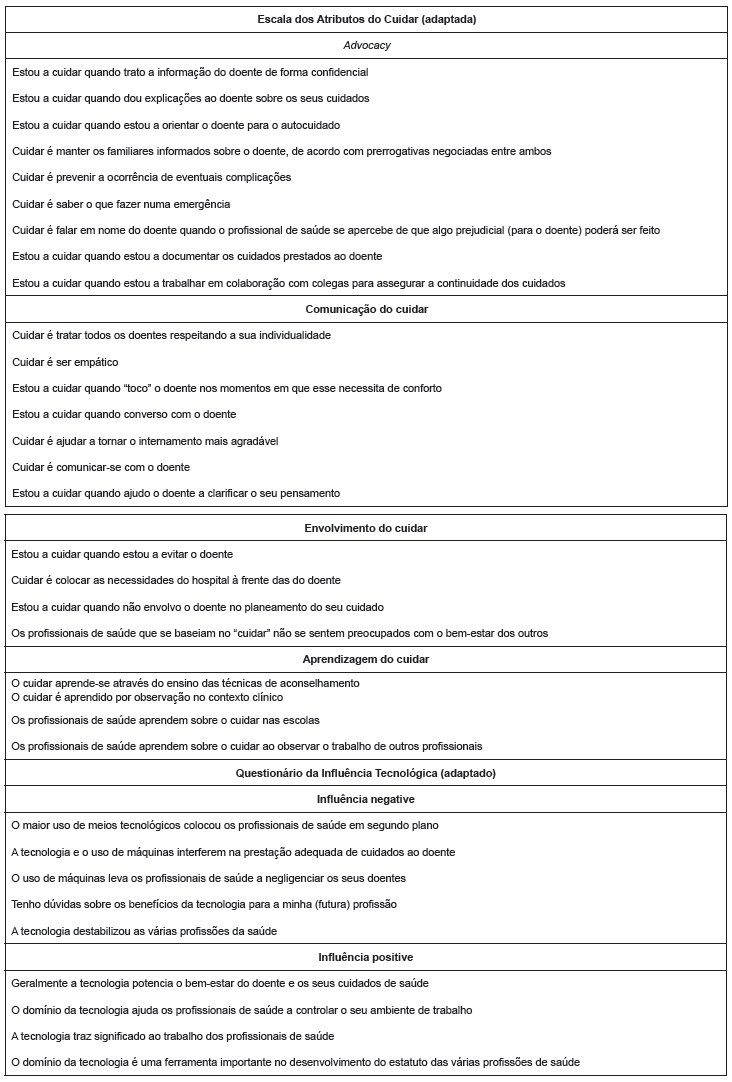
- Final distribution of items among the scale dimensions

After analyzing the results, the initial psychometric properties of the translation and adaptation of the Caring Attributes Questionnaire to the Portuguese language can be considered very reasonable, despite differences in the structure when compared to the original scale, immediately revealing the reduction in the number of items from 31 to 24 (Figure 1). The four factors from the international scale were maintained and, despite some migration among the items, the large majority is distributed in accordance with the initial version, although the causes of this difference cannot be fully explained. Nevertheless, it is important to highlight the cultural differences between the Portuguese population and the set of countries in the original study. In addition, which may be the most significant point, the present study sample is more heterogeneous since, as opposed to the first study, which only considered nurses, this sample included nurses, physicians and students from both areas. According to the author^(^ 9 ^)^, the scale tends to reveal lower alpha coefficients in more heterogeneous samples. As a result of the changes made, however, the alpha for the total scale was 0.848, close to the 0.88 of the original scale in the international sample.

As regards the Technological Influence Questionnaire (TIQ), after analyzing the presented results, the initial psychometric properties of the translation and adaptation of the Technological Influence Questionnaire to the Portuguese language can be considered very reasonable, despite differences in the structure when compared to the initial scale, immediately revealing the reduction in the number of items from 14 to 9 (Figure 1). In addition, as opposed to the original scale with a one-dimensional structure, in this study, an organization in two dimensions was found, with an apparent separation between positive and negative aspects. Once again, the cultural differences and the now more heterogeneous population seem to justify the differences found. These changes resulted in a Cronbach's alpha coefficient of 0.777 for the total scale, higher than the coefficient of the original scale (0.75).

## Conclusion

The validation process of the Caring Attributes Questionnaire and the Technological Influence Questionnaire started to construct a tool to study the relation between these two variables. After analyzing the results, it seems that this objective was positively achieved, as the scales demonstrate very reasonable psychometric properties. The analysis by the experts and the groups used shows that the tool is easy to understand and complete. The only less positive aspect was the loss of some original items, probably due to the heterogeneity of the selected population.

The validation of these scales entails the possibility of further studies on an essential aspect of health care, which is the relation between the technological influence and the health professionals' care. As mentioned in the introduction, different theoretical theses exist that appoint the technological influence as something negative for care delivery, but the few scientific studies that exist point in the opposite direction. Therefore, the validation of these tools and their general application will permit a deeper look into this theme.
